# Facile fabrication of B-rGO/ZnFe_2_O_4_ p–n heterojunction-based S-scheme exciton engineering for photocatalytic Cr(vi) reduction: kinetics, influencing parameters and detailed mechanism†

**DOI:** 10.1039/d4ra03049d

**Published:** 2024-06-25

**Authors:** Kundan Kumar Das, Upali Aparajita Mohanty, Lekha Paramanik, Dipti Prava Sahoo, Kulamani Parida

**Affiliations:** a Centre for Nanoscience and Nanotechnology, SOA (Deemed to be University) Bhubaneswar-751030 Odisha India paridakulamani@yahoo.com kulamaniparida@soa.ac.in +91-674-2581637 +91-674-2379425

## Abstract

The fabrication of p–n heterostructures was found to be an effective strategy to stimulate the interfacial exciton shipment and photocatalytic reactions. Herein, we report a p–n junction synthesized by combining p-type boron-doped reduced graphene oxide (B-rGO) with an n-type ZnFe_2_O_4_ semiconducting material for Cr(vi) reduction under LED light irradiation. The band structures of ZnFe_2_O_4_ and B-rGO were evaluated using UV-vis spectroscopy, Mott–Schottky (M–S) plots and photocurrent studies. The results indicated that ZnFe_2_O_4_ and B-rGO exhibit a conventional type-II charge transfer, and the Fermi-level (*E*_F_) of ZnFe_2_O_4_ was found to be much lower than that of the B-rGO material. Based on these investigations, an S-scheme charge-migration pathway was suggested and demonstrated by the photocatalytic activity and nitroblue tetrazolium (NBT) chloride experiments. The optimal 2 wt% B-rGO/ZnFe_2_O_4_ heterojunction exhibits the highest photocatalytic performance, *i.e.* 84% of Cr(vi) reduction in 90 min under 20 W LED light irradiation with a rate constant of 0.0207 min^−1^, which was 4.6- and 2.15-fold greater than that of ZnFe_2_O_4_ (ZnF) and B-rGO, respectively. The intimate interfacial contact, excellent photon-harvesting properties, effective exciton segregation and availability of active electrons are some factors responsible for enhanced photocatalytic Cr(vi) reduction. In order to fulfill the demand of applied waste-water management, the influences of various photocatalyst amounts, pH values and co-exiting ions on photocatalytic activities were evaluated. Finally, this work provides a way to fabricate S-scheme-based p–n-heterostructures for photocatalytic wastewater treatment.

## Abstract 2

The scheme represents the photocatalytic Cr (VI) reduction by 2BrZn p-n based S-scheme heterojunction photocatalyst.

## Graphical abstract

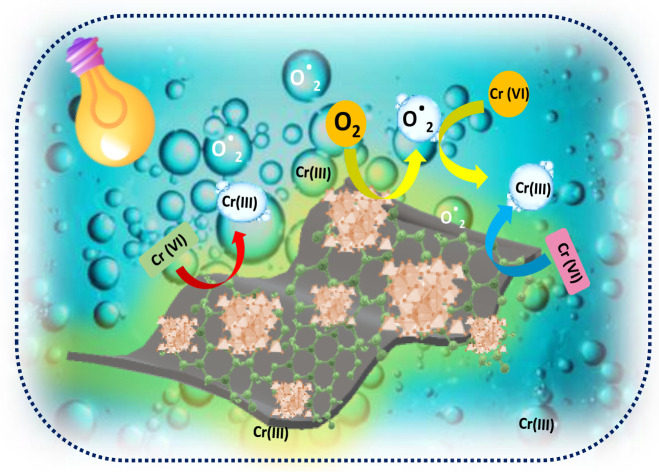

## Introduction

The accelerated growth of industrialization, globalization and high living standards have indirectly applied an incredible amount of pressure on the scientific community to resolve the environmental problems, specifically the water pollution across the sphere to a great extent. Water pollution caused by heavy metals is well known as the utmost hazardous due to their toxicity and eternity.^1–5^ Moreover, the solubility and persistence of these heavy metals in water bodies is a major concern as one-third of the global population are unable to access clean and affordable water for daily-life utilization.^6^ Among various heavy metals (As, Cr, Pb, Se, Hg and so-on), hexavalent chromium (Cr(vi)) is known as the most toxic pollutant and a serious threat to both human health and world ecological balance because of its incomplete eradication, high solubility, long-term stability, and nature of causing various deadly diseases such as cancers, genetic disorders, and lung malignancies. Metallurgy, chromium mining, electroplating, and petroleum refineries are some of the sources discharging this heavy metal into aquatic ecosystems.^7–10^ Therefore, it is highly necessary to find an instant solution to solve these problems; henceforth, the brainy minds of scientific society share all their knowledge, experience and intellect to alleviate these catastrophic problems for healthy future. The photocatalytic removal of this poisonous contaminant is considered as the most advanced approach from a green chemistry perspective.^11^ Therefore, abundant, sustainable and promising photocatalysts are in high demand, and their design and development is necessary. In this context, our group has reported some works in photocatalytic Cr(vi) reduction. Patnaik *et al.* has designed a Au-Pd-g-C_3_N_4_ composite for Cr(vi) reduction and found that the fabricated material achieved 56% of Cr(vi) reduction under visible light irradiation for 2 h.^12^ Similarly, Sahu *et al.* synthesized Cd–Mo–Se alloyed quantum dots for Cr(vi) reduction, which displayed 93.6% reduction in 2 h of irradiation.^13^ Das and his team members fabricated ZnFe_2_O_4_-Al_2_O_3_-MCM-41 by a wet-impregnation method for photocatalytic Cr(vi) reduction and exhibited 66% reduction in 60 minutes of sunlight irradiation.^14^

Encouragingly, semiconductor-based photocatalysis is the most promising and sustainable technology developed so far, which has the ability to solve all these problems. In this pathway, zinc ferrite (ZnFe_2_O_4_), an n-type semiconductor photocatalyst, has attracted extensive attention due to its excellent physicochemical properties such as narrow band gap (1.6–2.1 eV), wide visible light absorption range and tremendous photochemical stability.^14,15^ However, its photocatalytic activity is very low specifically towards photocatalytic Cr(vi) reduction due to inconvenient segregation of excitons, shorter hole diffusion distance and poor conductivity. Therefore, designing photocatalysts with prerequisite characteristics is the need of the hour. In order to achieve this, various alternative strategies were developed and explored, but among them, the p–n heterostructure strategy has gained significant interest and has been found to be quite fruitful. The work-function difference between p- and n-type materials not only enhances the photon absorption ability but also toddles the heterostructure to acquire the potentials, accelerating the transmission of excitons in both opposite pathways due to the creation of a built-in electric field at the interfacial junction, which ultimately improves the exciton segregation process.^16–18^ Sun and his members designed a BiOCl/ZnFe_2_O_4_ p–n-based system for RhB degradation under photon illumination.^18^ Guo and co-workers prepared a Ag_2_O/ZnFe_2_O_4_ p–n junction hydrothermally for BPA degradation.^19^ Similarly, Zhou and his group members synthesized ZnFe_2_O_4_/SnS_2_ p–n heterojunctions for methyl orange degradation under visible-photon irradiation.^20^ Therefore, more investigations are required to design a benchmark ZnFe_2_O_4_ framed p–n heterojunction system. In search of a better counterpart to ZnF, the 2D based-carbon allotropes were found to be the best choice. In this regard, boron-doped reduced graphene oxide (B-rGO) displaying p-type behaviour was investigated as a suitable photocatalyst due to some unique features such as high specific surface area, controllable interfaces, and high carrier mobility.^21^ Although the excitons are well separated through p–n heterojunction systems, they still exhibit some shortcomings from the kinetics, dynamic and thermodynamic viewpoints. To this end, Yu and his group members developed a novel step-scheme (S-scheme) heterojunction consisting of oxidation and reduction photocatalysts with staggered band-alignment.^22^ The S-scheme heterojunction possesses multiple supremacies such as proficient charge separation, quenching nonsensical reductive electrons and oxidative holes, while utilising powerful electrons and holes. The driving force for the charge transfer pathway in S-scheme is the generation of internal electric field (IEF), and hence, it is essential to generate an internal electric field between the photocatalysts for directional charge transfer.^23–26^ However, to the best of our knowledge there is no such article concerning B-rGO/ZnFe_2_O_4_ with built-in p–n heterojunctions following the S-scheme charge transfer dynamics.

Inspired by this, in this work we successfully fabricated a series of B-rGO/ZnFe_2_O_4_ p–n heterojunctions following the S-scheme charge transfer dynamics by a hydrothermal method. The synthesized photocatalysts were studied for the photo-reduction of Cr(vi) contaminant. The photocatalytic activity of the material was investigated under various operational parameters to find out their impact along with the detailed kinetics study. Further, the fabricated nanohybrid exhibits significant enhancement in activity corresponding to the effective exciton segregation and higher reduction potential excitons available through S-scheme charge transfer after the creation of p–n heterojunction. The proposed S-scheme charge dynamics was well supported by the photocatalytic activity. Similarly, the p–n heterojunction was verified by the photocurrent density and M–S measurements. The scavenger study with NBT experiments and photocatalytic activity proved that the p–n heterojunction follows the S-scheme charge transfer pathway.

## Experimental

### Materials

Zinc-nitrate hexahydrate [Zn(NO_3_)_2_·6H_2_O] (98%), ferric nitrate nonahydrate [Fe(NO_3_)_3_·9H_2_O] (98%), *tert*-butyl alcohol (TBA) (99%), silver nitrate (AgNO_3_) (99%), citric acid (CA) (99%), benzoquinone (BQ) (98%) and NH_3_ were purchased from Merck, India. Graphite powders and KMnO_4_ were obtained from Sigma-Aldrich. All reagents were pure and of analytical grade, and can be used directly.

### ZnFe_2_O_4_ (ZnF)

Hydrothermal techniques in combination with a calcination method were employed to synthesize the nanoparticles of ZnFe_2_O_4_ (ZnF). In a typical procedure, 2.5 mmol Zn(NO_3_)_2_·6H_2_O was mixed with 5 mmol Fe(NO_3_)_3_·9H_2_O in 60 mL of double-distilled water under continuous stirring to form a homogeneous solution at room temperature. Then, an aqueous solution of NH_3_ was introduced into the solution in order to maintain the pH of the solution at 12. The solution with the adjusted pH was transferred to a 100 mL Teflon and the container was sealed and maintained at 180 °C for 12 h. After the reaction was over, the container was ventilated to ambient-room conditions. The obtained product was cleansed with an aqueous ethanol solution and finally dried overnight at a temperature of 80 °C. After drying, the product was ground into powder, finally treated at 550 °C for 5 h in a muffle furnace and a brown-colored ZnF nanoparticle was obtained.^25^

### Synthesis of B-rGO/ZnFe_2_O_4_ (BrZn)

At first, graphene oxide (GO) was synthesized by an adapted Hummers method as previously reported in the literature.^27^ The B-rGO/ZnF composite material was fabricated by a hydrothermal technique. Initially, different quantities of GO (1, 2, and 4%) were added to a beaker containing 60 mL double-distilled water. Thereafter, boric acid (9.6 mmol) was added to the GO dispersed solution and kept under continuous stirring for next 2 h. Then a certain amount of ZnF was added to the above solution, stirred for another 1 h and finally transferred to an autoclave and maintained at 180 °C for 12 h. After the reaction was over, the container was cooled down naturally to room temperature. The obtained slurry was washed repeatedly with an aqueous ethanol solution and finally dried in an oven at 80 °C for further use.

## Results and discussion

**Fig. 1:**
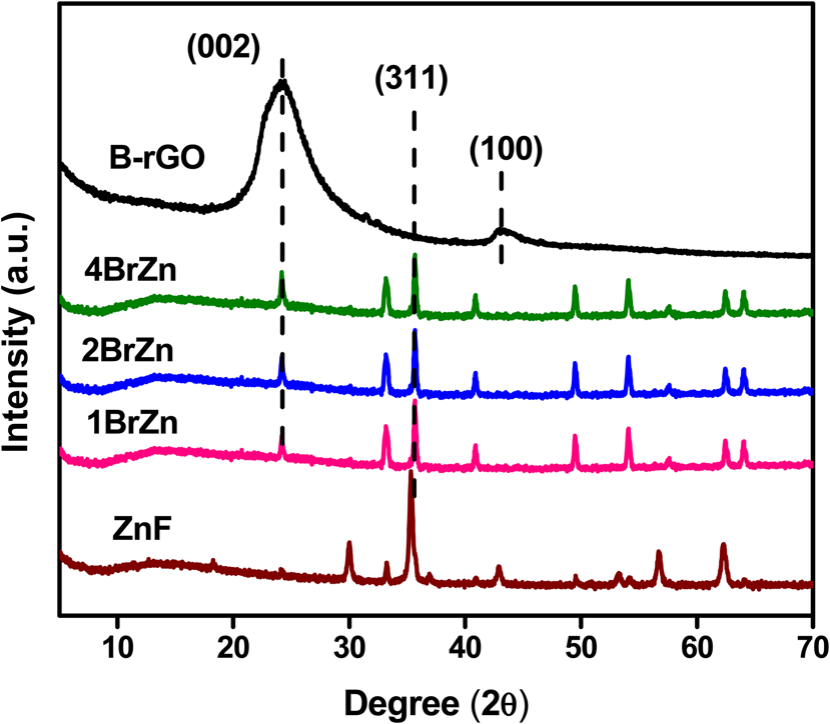
XRD diffractions of ZnF, B-rGO, 1BrZn, 2BrZn, and 4BrZn.

Powder X-ray diffraction (PXRD) measurements were employed to explore the phase purity, crystallinity and structural characteristics of the as-synthesized photocatalytic specimens, as demonstrated in Fig. 1. The diffraction signal of ZnF obtained at a 2*θ* value of 25.4° was attributed to the (311) plane, which was consistent with the cubic-phase of spinel ferrites according to JCPDS 01-077-0011.^25,28^ Similarly, the trademark signal of GO located at 10.1° was completely vanished to produce an additional diffraction at a 2*θ* value of 24.5°, which imitates the (002) plane of B-rGO. This explains about good exfoliations and complete reduction of GO sheets into B-rGO nanosheets with graphite-like crystallinity and less functional groups.^29,30^ The diffraction pattern of GO is provided in Fig. S1.† The (002) plane of B-rGO also signifies the amorphous characteristic of the material, which well matched with the SAED result, as discussed and provided in the later section. Moreover, a weak diffraction was noticed at 43.2° corresponding to the (100) plane, confirming the formation of turbostratic graphitic carbon.^31^ These two trademark diffractions of boron-doped rGO are recurrently discussed, verifying that our outcome is in good agreement with the results reported in the literature. The diffractions of composite materials, *i.e.* the BrZn p–n heterojunction was found to be similar to that of pristine ZnF along with the introduction of a new signal positioning at 23.5°, which can be further ascribed to the (002) plane of B-rGO. The appearance of both B-rGO and ZnF peaks in the as-synthesized BrZn composites proved the successful formation of heterojunctions among them. Furthermore, the diffraction intensity of the (002) plane of B-rGO was perceived to be enhanced with the increase in B-rGO amount in the BrZn heterojunctions. The introduction of B-rGO results in a positive shifting of the (311) plane of ZnF in the BrZn specimens along with a slight reduction in peak intensity. These outcomes and interpretations suggest that a strong interaction exists between ZnF and B-rGO during the synthesis process. The average crystallite size (*D*_*hkl*_) and average micro-strain of ZnF, B-rGO and BrZn composite materials were calculated using expression (1) and (2):^32^1
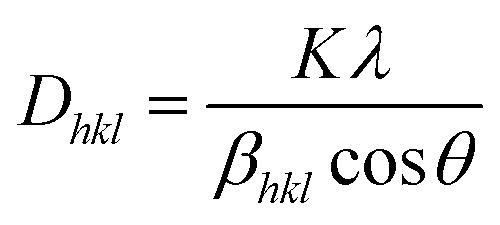
2
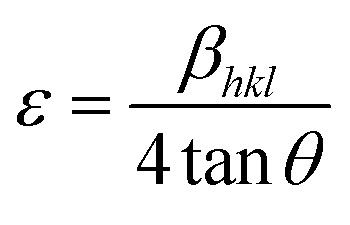
where *D*_*hkl*_ is the crystallite size in nm, *λ* is the wavelength of photons used in nm, *θ* is Bragg's angle, *β*_*hkl*_ is the wideness of the diffraction-beam (rad), and *K* is the shape-parameter of crystallites (*K* = 0.9, when *β*_*hkl*_ is defined as the half-high width of diffractions). Further, the inter-layered space (*d*) was calculated using Bragg's equation, where *λ* is the wavelength of incident X-ray photons (nm), and *n* and *θ* are the order and angle of diffractions respectively.^32^

3 *nλ* = 2*d* sin *θ*

At last, the lattice parameter for the cubic structure (*a* = *b* = *c*) was evaluated by adopting the expression and here *h*, *k* and *l* are the Miller indices and *d* is the inter-layer spacing.^33^4
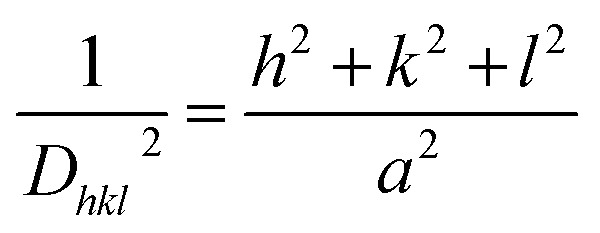


The average crystallite size, average micro strain, and inter-layer spacing, lattice parameters, and cell volume of the materials along with the lattice planes (311) and (002) for ZnF and B-rGO are tabulated in Table 1.

**Table 1:** Average crystallite size, micro strain and interlayer spacing of ZnF, B-rGO, 1BrZn, 2BrZn and 4BrZn

Sample	Average crystallite size (nm)	Average micro strain (1 × 10^−2^ lines per m^2^)	Interlayer spacing, *d* (in nm)		Lattice parameters (*a* = *b* = *c*, cubic system) (Å)		Unit cell volume *V* (Å)^3^	
			(311)	(002)	(311)	(002)	(311)	(002)
ZnF	26.5	1.6 × 10^−3^	1.12	—	5.68	—	82.39	—
B-rGO	14.2	2.3 × 10^−3^	—	2.56	—	6.89	—	52.46
1BrZn	22.4	1.8 × 10^−3^	1.48	1.55	4.19	8.08	73.77	52.49
2BrZn	20.1	1.6 × 10^−3^	1.5	1.53	4.16	8.12	72.56	53.12
4BrZn	19.7	1.5 × 10^−3^	1.52	1.49	3.98	8.17	71.67	53.58

**Fig. 2:**
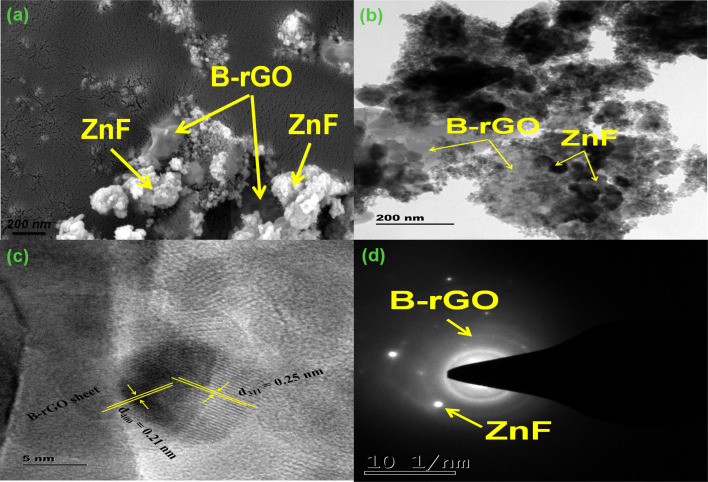
(a) FE-SEM image, (b) TEM image, (c) HR-TEM image, and (d) SAED patterns of the 2BrZn photocatalyst.

The inner and peripheral topological features of the fabricated 2BrZn composite were investigated through SEM and TEM characterizations. Fig. 2a illustrates the FE-SEM images of 2BrZn, which reveal that the nanoparticles of ZnF are well distributed all over the B-rGO sheets. The SEM images also visualize that the agglomerated particles of ZnF are well attached to the smooth surfaces of B-rGO sheets, endorsing a close interfacial heterojunction between ZnF and B-rGO which may enhance the photo-produced charge diffusion under visible-light irradiation. The EDS analysis and the elemental mapping as represented in Fig. S2† confirm the existence of Zn, Fe, B, and O elements in the 2BrZn specimen, illuminating the pureness of the material. Moreover, the good distribution of ZnF nanoparticles over the thin, porous and smooth sheet-like structure of B-rGO can be observed from the TEM images, as illustrated in Fig. 2b, specifying that the nanoparticles of ZnF are accumulated on B-rGO nanosheets to form an intimate hybridized 2BrZn binary heterojunction, which is in good agreement with the FESEM image. Moreover, the tiny nanoparticles have aggregated with each other to form large nanoparticles of ZnF, which can be evident from the pictorial image. The *d*-spacing provided in Fig. 2c of 0.26 nm was ascribed to the (311) lattice plane of cubic ZnF along with a distinct B-rGO area. However, the lattice spacing of B-rGO is absent because of its porous and electron sensitive nature. The SAED pattern of 2BrZn depicted in Fig. 2d illustrates various bright spots for ZnF along with the translucent rings of B-rGO, indicating the polycrystalline nature of the binary material.

**Fig. 3:**
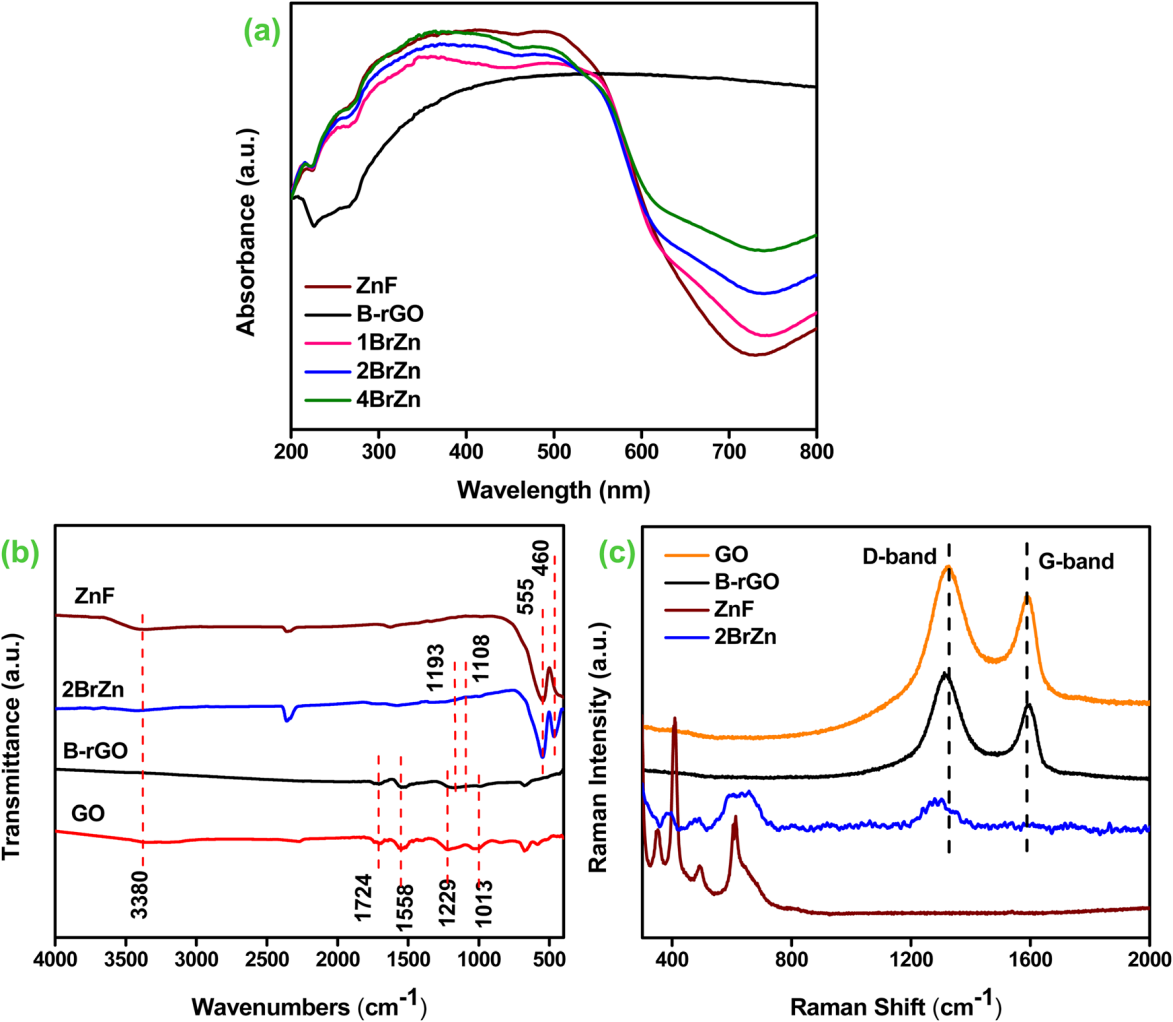
(a) UV-vis DRS spectra (ZnF, B-rGO, 1BrZn, 2BrZn, and 4BrZn). (b) FTIR transmittance (ZnF, GO, B-rGO, and 2BrZn). (c) Raman measurements (ZnF, GO, B-rGO, and 2BrZn).

The photophysical characteristic of the as-synthesized specimens is an essential parameter for examining the photocatalytic activities. Fig. 3a displays the optical absorption spectra of ZnF, B-rGO, 1BrZn, 2BrZn and 4BrZn composite materials. The B-rGO sample exhibits very strong absorption starting from the UV region to the IR region, which can be attributed to the black color of the sample. Moreover, a small absorption band was noticed at 260 nm for B-rGO, which is due to the n–π transitions. Similarly, a wide range of absorption was encountered for the spinel ZnF because of the existence of the Fe 3p ion. The principal cause behind the broad-absorption is the photo-sensing characteristic feature of Fe 3p ions along with the charge migration, which occurs among the oxidation numbers of iron (Fe 3p and Fe 2p) within the crystal lattice of ZnF.^14,34^ The charge migration between Zn–O and Fe–O ion was further identified by the appearance of a small absorption band between 200 and 300 nm wavelength. However, on close examination, two absorption bands can be observed for ZnF at 270 and 280 nm corresponding to the migration of electrons from oxygen to iron species.^34,35^ Furthermore, a strong absorption band-edge was noticed at 700 nm corresponding to the transfer of excitons from *E*_VB_ to *E*_CB_. However, the modification of ZnF with the introduction of B-rGO does not hamper the nature of the absorption spectra, rather it results in the bathochromic shifting of absorption-edge because of the presence of black colored graphene moieties. As the concentration of B-rGO increases, the photon absorbing capacity also increases, as evident from the rising absorption tail, and also enhances the photocatalytic effectiveness of ZnF.

FTIR spectroscopy analysis was carried out to investigate the existence of various functional clusters associated with their stretching and bending bonds of the as-prepared photocatalyst, and the obtained results are illustrated in Fig. 3b. For ZnF, two vibrations were observed at 435 and 554 cm^−1^ corresponding to the stretching and breathing vibrations of Zn–O and Fe–O bonds respectively.^14^ Similarly, B-rGO exhibits two supplementary peaks alongside the featured stretching vibration of GO, as previously reported in the literature.^21^ The two extra peaks were located at 1107 and 1182 cm^−1^ assigned to the B–C stretching and B–O bond respectively.^35,36^ As compared to GO, a stretching peak positioned at 3422 cm^−1^ gets completely vanished in the case of B-rGO, indicating that an alternation in the chemical structure occurred while moving from GO to B-rGO as reduction accelerates the shrinking of O–H functional groups.^21^ After the fabrication of 2BrZn, the wideness of the –OH stretching peak slightly reduces, indicating a strong interaction between B-rGO and ZnF. Moreover, a shifting in the featured peak of ZnF was observed in the IR absorption band of the heterojunction sample, which again suggests a strong interaction between the ZnF and B-rGO samples. In the 2BrZn sample, the manifestation of stretching and bending peaks of respective sample verifies the co-existence of both the materials, which advocates that the structural integrity of ZnF is maintained with B-rGO during the synthesis process.

Moreover, the presence of functional species and the conversion of GO into B-rGO were verified by Raman measurement. Fig. 3c portrays the Raman measurements of ZnF, GO, B-rGO and 2BrZn samples. The Raman signal of ZnF signifies that it belongs to the spinel family, which is consistent with XRD results. ZnF is supposed to display five Raman signals, but in this study, we are able to identify three signals. The Raman signals positioned below 610 cm^−1^ correspond to the symmetrical vibration of ferrite materials. Moreover, the Raman peaks beyond and underneath 600 cm^−1^ indicate the existence of tetrahedral and octahedral groups.^37^ The Raman measurement of GO represents that two solid peaks situated at 1322 and 1533 cm^−1^ correspond to ‘D and G’ signals respectively. The ‘D’ signal symbolizes the bending modes of the A1g symmetry, whereas the ‘G’ signal denotes the sp2 hybridized carbon framework.^31,38^ Further, the transformation of GO into the B-rGO was perceived from both shifting and reduced intensity of ‘D and G’ signals respectively. Moreover, the amplified *I*_D_/*I*_G_ ratio also notifies us about the transformation of GO to B-rGO. The peak-shifting is related to the structural abnormalities of graphene moieties, which resulted from the variation in bond distances and hole-donating features of the boron atom to heterojunction networks.^39,40^ The Raman measurement of the 2BrZn specimen preserves both the D and G bands of the graphene material supplemented with the basic Raman modes of ZnF, indicating the successful fabrication of the heterojunction material. Moreover, a shifting in D-band was observed for the 2BrZn material (Fig. 3c), which again specifies about the strong interaction between ZnF and B-rGO specimens.

**Fig. 4:**
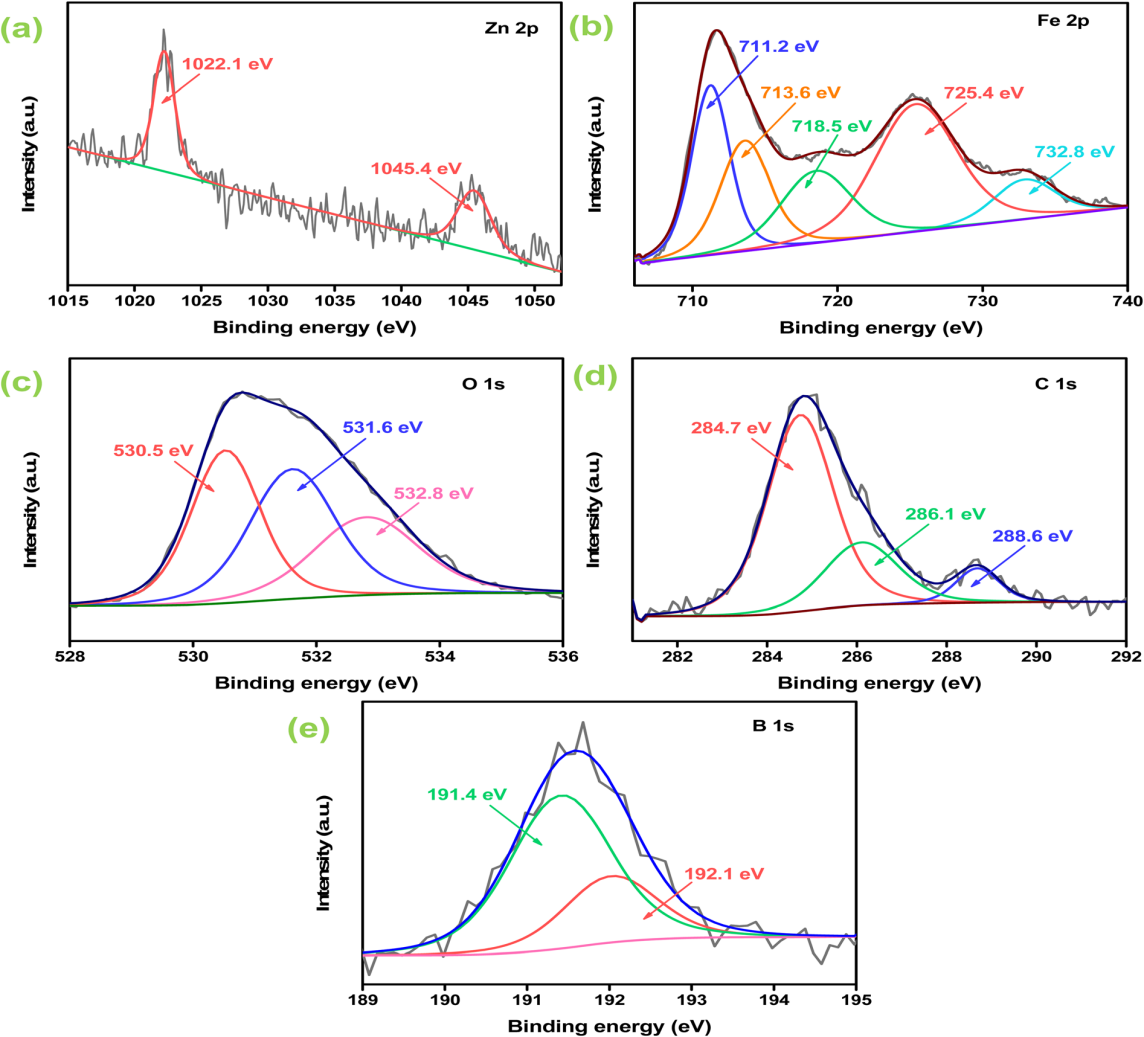
High-resolution XPS spectrum of 2BrZn: (a) Zn 2p; (b) Fe 2p; (c) O 1s; (d) C 1s; and (e) B 1s.

Furthermore, X-ray photoelectron spectroscopy (XPS) measurement was performed to gain information related to the atomic-bonding, surface chemical states and electronic environment of the as-synthesized 2BrZn specimen. As represented in Fig. S3,† the wide-scan spectrum tends to retain the foremost peaks of Zn, Fe, O, C and B elements. However, a weak signal was noticed for the B element, which may be because of the loading of low concentrations of B-rGO specimen. Further, no additional peaks were detected in the survey spectrum, indicating the purity of the specimen also observed in XRD and EDX results. The Zn 2p spectrum displays two clear peaks at 1022.1 and 1045.4 eV corresponding to the 2p_3/2_ and 2p_1/2_ spin states, which confirms that Zn exists in +2 oxidation state (Fig. 4a).^41^ Similarly, the Fe 2p spectrum highlights two distinct signals at 711.2 and 725.4 eV corresponding to Fe^3+^ 2p_3/2_ and Fe^3+^ 2p_1/2_ along with two satellite peaks positioned at 718.5 and 732.8 eV respectively (Fig. 4b).^42^ Further, the Fe 2p_3/2_ spin-state was deconvoluted into two peaks in order to enlighten the detailed characteristic features of Fe^3+^ oxidation states in the 2BrZn specimen. The deconvoluted signals at 711.2 and 713.6 eV can be ascribed to the tetrahedral and octahedral sites respectively.^14^ From the above-mentioned findings, it was concluded that Fe is present in the Fe^3+^oxidation state as the binding energy for Fe^2+^ oxidation state is about 709.5 eV, which is well below the binding energies of our obtained oxidation state.^28^Fig. 4c illustrates the O 1 s XPS spectrum with three deconvoluted signals located at 530.5, 531.6 and 532.8 eV. The signal positioned at 530.5 eV represents Fe–O–C providing information about the good interaction between Fe and carbon species present in 2BrZn,^28^ whereas the peak centered at 531.6 eV can be assigned to the oxygen-containing species (epoxy and hydroxyl) and that situated at 532.8 eV corresponds to the absorbed oxygen groups.^15^ The appeared signals of O 1s spectrum basically originate from ZnF and leftover oxygen groups from B-rGO. Fig. 4d depicts the C 1s XPS spectrum with four deconvoluted peaks centered at 284.7, 286.1 and 288.6 eV corresponding to the adventitious C–C/C=C bonding of aromatic rings, C–O bond of alkoxy groups and O–C=O species respectively.^28,43^ The presence of boron element in the 2BrZn specimen was verified by the B 1 s spectrum. As highlighted in Fig. 4e, B 1s was split into two distinguishable signals located at 191.4 and 192.1 eV respectively. The signal at 191.5 eV can be assigned to the B–C bond corresponding to the substitution of graphitic BC_3_ in the graphene nanosheet and the peak at 192.1 represents the B–O bonding in BC_2_O, which substantiates the successful doping and interaction of boron atoms in the graphitic backbone.^38,44^

**Fig. 5:**
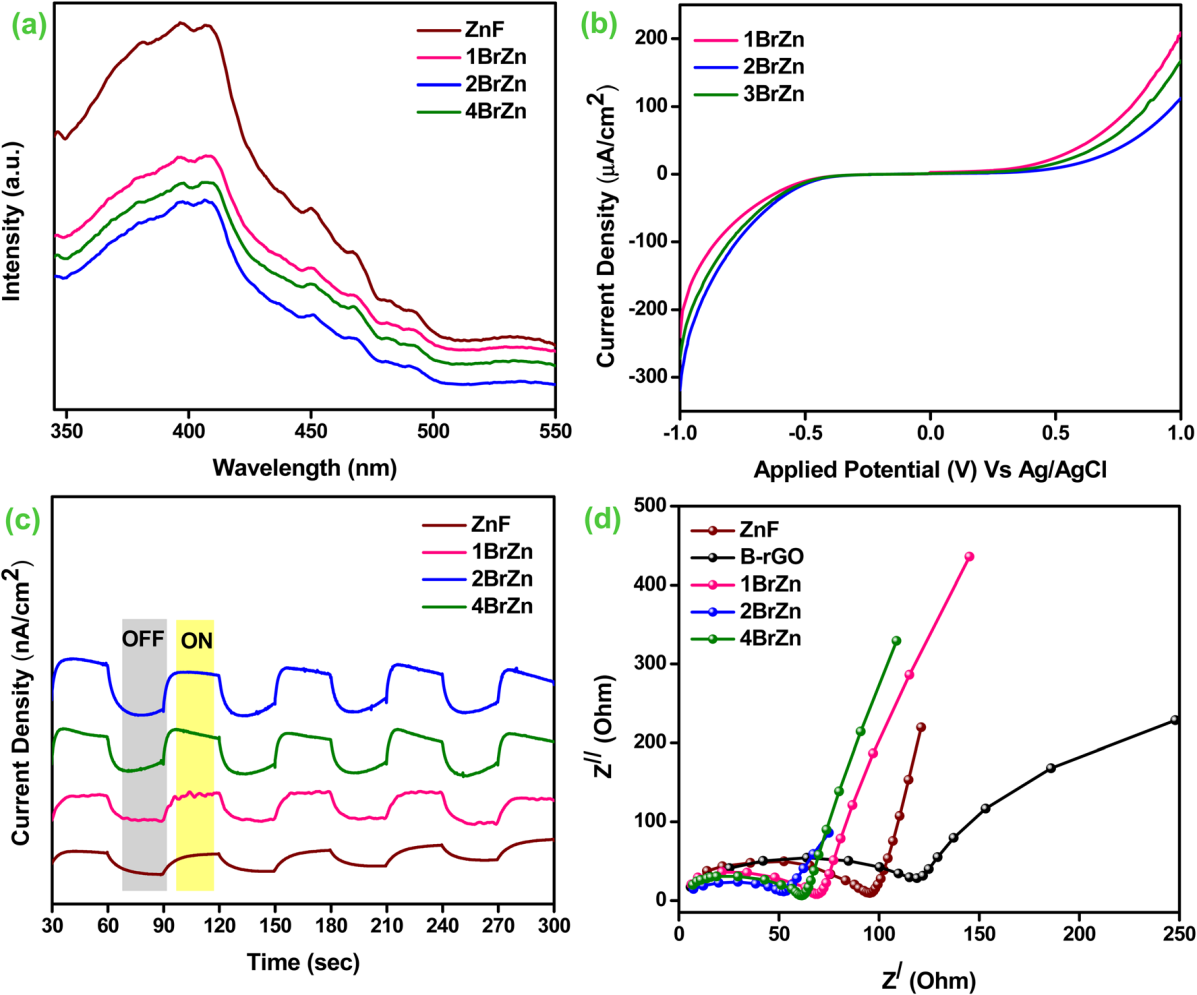
(a) Photoluminescence spectra (ZnF, 1BrZn, 2BrZn, and 4BrZn). (b) Photocurrent densities (1BrZn, 2BrZn, and 4BrZn). (c) Transient photocurrent (ZnF, B-rGO, 1BrZn, 2BrZn, and 4BrZn). (d) EIS spectra (ZnF, B-rGO, 1BrZn, 2BrZn, and 4BrZn).

Furthermore, to evaluate the exciton dynamics of photocatalytic specimens, the photoluminescence study, photo-current measurements, transient photocurrent and EIS analysis were employed. It is well known that the luminescence spectrum arises from the recombining process of photo-produced charge carriers. The obtained luminescence spectrum is directly proportional to the pace e^−^/h^+^ separation process, *i.e.* a highly intense spectrum describes the poor separation, whereas a low intense spectrum explains better and fruitful separation.^45^Fig. 5a illustrates the photoluminescence spectra of the as-synthesized specimens, which have been excited at a wavelength of 325 nm. The parent ZnF material illustrates a sharp and strong near-band edge signal at 396 nm corresponding to the direct recombination of photo-excitons in the photocatalyst. However, after the introduction of B-rGO, a similar nature of luminescence peak with reduced intensity was obtained for the 1BrZn, 2BrZn, and 4BrZn composite materials, suggesting that the addition of an sp^2^ framework in the form of black-colored B-rGO to the ZnF lattice delays the recombination process of e^−^/h^+^ pairs by transferring the charge-carriers through S-scheme-based p–n heterojunctions. Among the heterojunctions, 2BrZn displays the lowest luminescence spectrum indicating the superior segregation and transmission of excitons by hindering the recombination process of photo-exciton, which is a key factor responsible for heightened photocatalytic performances.

Moreover, the separation and shipment of photo-excitons in the heterojunctions were further corroborated by photoelectrochemical experimental studies. Basically, it is known that the larger the amount of photocurrent data, superior is the separation process of photo-produced charge-carriers. Fig. S4† demonstrates the photocurrent measurement of ZnF and B-rGO materials under photon illumination. Upon exposure to photons, the ZnF electrode produces a current of 337 μA cm^−2^ at +1 V *vs.* Ag/AgCl. Similarly, the B-rGO specimen generates the current starting from almost negligible at 0 V and continued to 109 μA cm^−2^ at a scan of −1 V Ag/AgCl. Fig. 5b displays the photocurrent of 1BrZn, 2BrZn and 4BrZn composite materials. In comparison to ZnF, an inferior amount of photocurrent was observed for all composite materials in the upward direction. Similarly, as compared to B-rGO, the composite material exhibits expressively superior photocurrent in the downward direction. This type of photocurrent behavior indicates the development of p–n heterostructures between B-rGO (p-type) and ZnF (n-type), co-related with the M-S analysis and also informs about the effective charge separation, which occurs through S-scheme charge transfer. In particular, the 2BrZn photocatalyst was found to be the best among all heterojunction materials, where a forward current of 112 μA cm^−2^ was attained, which was nearly 3 folds lower than that of the parent ZnF at a scan of +1 V whereas a backward current of 318 μA cm^−2^ was achieved, nearly 3-fold greater than that of parent B-rGO at potential scan of −1 V. This photocurrent density of 2BrZn can be attributed to the effective charge segregation and facile anti-recombination due to the S-scheme-based p–n heterojunction. In this current study, a proper channelization of e-s and h+ between CB_ZnF_ and VB_B-rGO_ occurs due to the formation of the S-scheme charge migration pathway that results in the enhancement of p-type and detraction of n-type photocurrent respectively.

Further, we performed the transient photocurrent measurements to verify the separation, shipment and conversion efficiency of charge carriers. Basically, the photocurrent responses were produced due to the build-up and decay of charge concentrations reaching the surface. However, a reversible current was perceived for all semiconducting materials accompanied by the interchanging on-off photon cyclic experiments for 240 s time period with a 30 s interval. As displayed in Fig. 5c, it was clearly observed that the 2BrZn p–n heterojunction exhibited the highest photocurrent density, which revealed of having maximum concentration of reducing e^−^s cloud for the photocatalytic reaction in comparison to parent ZnF and other p–n heterojunctions, which can be credited to the effective segregation of photo-excitons through the S-scheme pathway after the establishment of p–n junctions and also informs that the composite material was more sensitive to photon irradiation. These outcomes establish that B-rGO could acts as an electron trapper to hinder the backward movement of electrons to ZnF, thereby suppressing the exciton recombination process. Under photon irradiation, the generation and separation of photo-excitons occurs from where the electrons move to the bulk and holes move to the surface of the semiconductor. Before attaining the steady-state condition, the upraise-decay dynamic of transient-photocurrent delivers valuable knowledge regarding the trapping and de-trapping of photo-excitons.

Further, the electrochemical impedance spectroscopy (EIS) techniques for the as-synthesized materials under photon irradiation are demonstrated in Fig. 5d. The investigation offers a valuable understanding of the movement of charge-carriers, and conductivity and resistivity of the material during the photocatalytic reaction related to the electrode–electrolyte interface. Basically, the smaller the impedance arc radius, the higher the carrier mobility and *vice versa*, whereas at a higher frequency, the inclined straight-line attributes to the Warburg impedance towards the ion-diffusion process. Fig. 5d illustrates the electrochemical impedance spectra of the synthesized materials, and the size of the Nyquist semicircular arc was in the following sequence: B-rGO, ZnF, 1BrZn, 2BrZn and 4BrZn composite materials. The smallest Nyquist arc was obtained for 2BrZn, which suggests superior channelization of photo-excitons through the p–n heterojunction-based S-scheme charge transfer pathway. The introduction of B-rGO also reduces the charge transfer resistance demonstrating that the S-scheme-based p–n heterojunction had a faster rate of exciton shipment, which was responsible for enhanced photocatalytic performances. Subsequently, the outcomes of the above characterizations verify that the composite materials retain a strong charge transfer capability, which delays the number of excitons compounding and hence displays elevated photocatalytic activity of Cr(vi) reduction.

### Photo-reduction of Cr(vi)

**Fig. 6:**
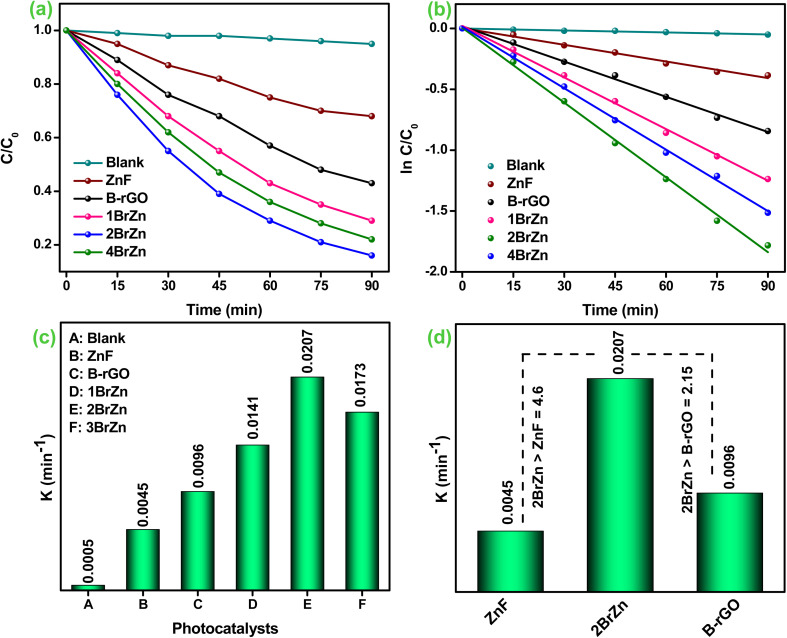
(a) Cr(vi) reduction curves. (b) Corresponding pseudo-first-order curves. (c) Apparent rate-constant *K* values (ZnF, B-rGO, 1BrZn, 2BrZn, and 4BrZn respectively). (d) Comparison of the *K* value of 2BrZn with those of ZnF and B-rGO.

The photocatalytic ability of the synthesized specimens was explored by Cr(vi) reduction under LED photon irradiation. The photoreduction profiles of 20 ppm Cr(vi) under various conditions were determined, and the obtained results are portrayed in Fig. 6. The blank experiments were performed to determine the photolysis property of Cr(vi), which can be overlooked as Cr(vi) is hardly transformed to Cr(iii) deprived of a photocatalyst. Similarly, the existences of only photocatalyst have no impact in the reduction process and it was observed that either photolysis or photocatalyst alone attains any significant reduction of Cr(vi). To our utter surprise, an augmentation in Cr(vi) reduction was perceived after the introduction of a reaction medium under LED light irradiation. Before illumination, the aqueous solutions of Cr(vi) along the photocatalyst were continuously stirred in a shady environment for 30 minutes to accomplish the adsorption–desorption equilibrium. Fig. 6a portrays the removal efficiency of Cr(vi) pollutant over the as-synthesized specimens, and it was noticed that the binary specimen illustrates better photocatalytic performances than those of the parent specimen. The rate of 20 ppm Cr(vi) reduction follows the order 2BrZn > 4BrZn > 1BrZn > B-rGO > ZnF respectively. The highest Cr(vi) reduction was obtained for 2BrZn, *i.e.* 84% in 90 minutes of illumination, which is about 4.6- and 2.15-fold greater than that of ZnF and B-rGO respectively corresponding to the following reasons. First of all, the construction of a p–n heterojunction provides highly dispersive active sites for the photo-reduction of Cr(vi). Second, the p–n junction creates an internal electric field (IEF) followed by the S-scheme charge-transfer pathway that facilitates the charge separation and migration at the interface and retains robust reductive and oxidative reaction competences.

Fig. 6b displays the heightened photocatalytic activities over different fabricated samples and the entire sample tends to follow a pseudo-first-order pathway, which is confirmed using eqn (5):5
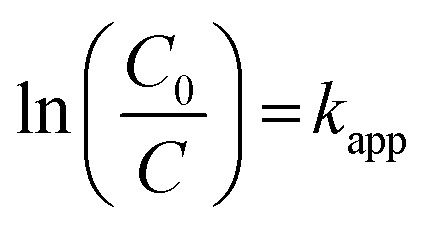


Further, the aforementioned expression was again utilized for assessing the half-life time (*t*_1/2_). Basically, the half-life time is the amount of time required for Cr(vi) reduction into its half-concentration from its original concentration and is demonstrated using expression (6):

6 *t*_1/2_ = 0.693/*k*_app_

The rate-constant (*k*_app_), co-efficient factor (*R*^2^), and half-life value (*t*_1/2_) of Cr(vi) reduction are presented in Table 2. Fig. 6c demonstrates the rate-constant of all fabricated specimens, where the *K*_app_ value of 2BrZn was found to be 0.0207 min^−1^ which was much higher than that of blank, ZnF, B-rGO, 1BrZn, and 4BrZn, and the obtained values are presented in Table 2. The rate-constant for 2BrZn was 4.6- and 2.15-fold greater than that the pristine ZnF and B-rGO specimens (Fig. 6d). Additionally, the photocatalytic Cr(vi) reduction by 2BrZn was greater than that of the other reported systems, and the details are provided in Table S1.†

**Table 2:** Degradation efficiencies, *k*_app_, *R*^2^ and *t*_1/2_ values of various photocatalysts for photocatalytic Cr(vi) reduction

Photocatalyst	Cr(vi) reduction efficiency (%)	(*R*^2^)	(*k*_app_) (10^−4^ min^−1^)	*t*_1/2_ (min)
Blank	5	0.97	5	0.1386
ZnF	32	0.98	45	0.0154
B-rGO	57	0.99	96	0.0072
1BrZn	71	0.98	141	0.0049
2BrZn	84	0.99	207	0.0033
4BrZn	78	0.99	173	0.004

**Fig. 7:**
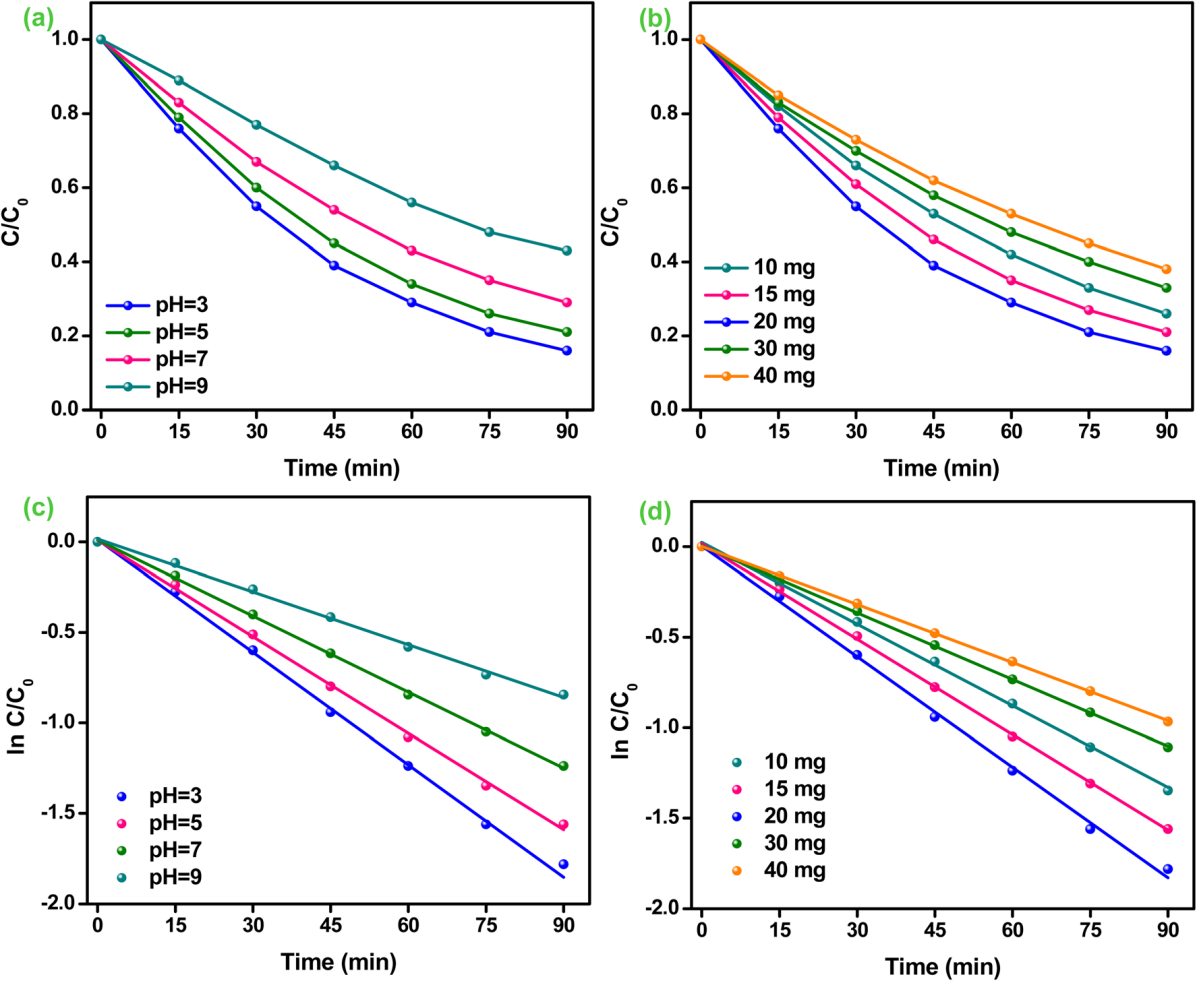
Effect of operational parameters: (a) pH; (b) photocatalyst amount; and (c and d) respective pseudo-first-order kinetics of the parameters of 2BrZn.

In the photocatalytic Cr(vi) reduction process, the initial pH was found to be a critical parameter as it affects the adsorption of chromium species on the photocatalyst surface. The synthesized 2BrZn photocatalysts exhibit a higher reduction activity in an acidic medium than in a basic medium (Fig. 7a). Under acidic conditions, the photocatalyst surface becomes positively charged due to protonation that improves the adsorption of HCrO^4−^ and Cr_2_O_7_^−^species. Henceforth, the highest Cr(vi) reduction was achieved at pH 3 (*i.e.*, 84%) in 90 minutes, in agreement with the results reported in the literature.^46^ On the contrary, under basic conditions, the adsorption of CrO_4_^2−^ is significantly repressed by the adsorption of OH^−^ ions on the surface of catalysts and henceforth reduces the activity to 22% at pH 9. The sequence of Cr(vi) reduction efficiency at various pH values follows the order of pH3 > pH5 > pH7 > pH9, as displayed in Fig. 7a. Under various pH values, Cr(vi) reduction follows the equations below:

In acidic media

7 Cr_2_O_7_^2−^ + 14H^+^ + 6e^−^ → 2Cr^3+^ + 7H_2_O

In neutral media

8 CrO_4_^2−^ + 8H^+^ + 3e^−^ → Cr^3+^ + 4H_2_O

In basic media

9 CrO_4_^2−^ + 4H_2_O + 3e^−^ → Cr(OH)_3_ + 5OH^−^

The dosage of photocatalysts also plays a vital part in the reduction process of Cr(vi). Fig. 7b depicts the impact of the photocatalyst amount on the conversion efficiency of Cr(vi) at 10 mg, 15 mg, 20 mg, 30 mg and 40 mg of 2BrZn. The proficiency of Cr(vi) reduction surges with the increase in the amount from 10 mg to 20 mg of the photocatalyst, inferring that increasing the catalyst amount could significantly enhance the activity. It is worth noting that the reduction process decreases with the further increase in the amount of photocatalysts, which can be due to the presence of restricted number of active sites on the catalyst surface. A secondary reason for reduced activity is related to the photon absorbing feature.^47^ The photon absorbing area of the photocatalyst is narrowed down if the dosage of photocatalyst surpasses the optimized figure. Therefore, 20 mg of the photocatalyst was regarded as the ideal dosage for Cr(vi) reduction. Moreover, the reduction kinetics of different parameters (pH and catalyst dosage) for Cr(vi) reduction tends to follow the pseudo-first-order model, as illustrated in Fig. 7c and d respectively. Basically, the polluted water is known to retain several inorganic ions that might hamper the photo-reduction process by competing with Cr(vi) ions for adsorption on the active sites of photocatalyst. Henceforth, the effects of co-existing ions such as Na^+^, Ca^2+^, Mg^2+^, SO_4_^2−^, CO_3_^2−^ and Cl^−^ on Cr(vi) reduction using the 2BrZn system were studied, and the results are portrayed in Fig. S5.† From the pictorial image, it can be observed that the Cr(vi) reduction was minutely repressed by these existing ions. The influence of Na^+^, Ca^2+^, and Mg^2+^cations on Cr(vi) reduction revealed that the cations have a negligible impact due to the existence of viscoelasticity between the catalyst surface and the bulk solution, which quashes the activity by impeding the adsorption of Cr(vi) (Fig. S5a†). Similarly, the impacts of SO_4_^2−^, CO_3_^2−^ and Cl^−^ ions were also studied, and it was found that Cl^−^ has an insignificant impact in the Cr(vi) reduction process. However, the reduction process was slightly inhibited by the existence of SO_4_^2−^ and CO_3_^2−^ ions, which may be attributed to their high negativity and competing with CrO_4_^2−^ ions for adsorption sites under neutral conditions (Fig. S5b†).^48^

**Fig. 8:**
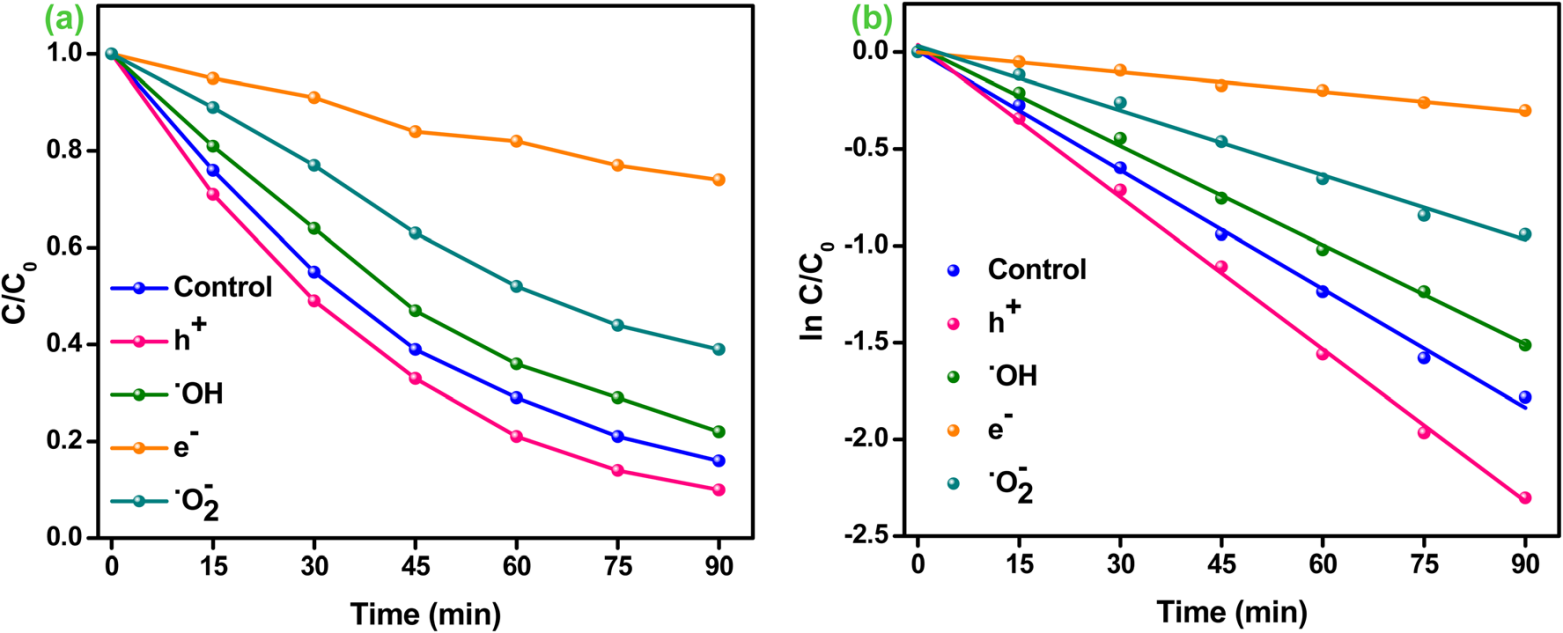
(a) Effect of different trapping reagents. (b) Kinetics of various trapping reagents for Cr(vi) reduction over the 2BrZn photocatalyst.

Moreover, the trapping experiments were carried out for finding the active species responsible for Cr(vi) reduction, and the results are shown in Fig. 8a. Citric acid (CA), *tert*-butyl alcohol (TBA), silver nitrate (AgNO_3_) and *para*-benzoquinone (p-BQ) were utilized as hole (h^+^), hydroxyl (OH^−^), electron (e^−^) and super-oxide (˙O_2_^−^) trapping reagents respectively.^1,16,45^ The reduction of Cr(vi) was considerably inhibited from 84% to 34% after the introduction of AgNO_3_, which confirms that electrons are the foremost reactive species. The introduction of AgNO_3_ was known to capture the e^−^s during the Cr(vi) reduction process, inhibiting the direct transformation of Cr(vi) to Cr(iii). Moreover, the reduction competence of Cr(vi) upsurges to 92% after the addition of CA into the reaction system, which may be due to the fact that CA consumes h^+^ during the reaction process, thereby decelerating the recombination's rate of excitons.^48^ Furthermore, the Cr(vi) reduction was slightly restricted after adding p-BQ into the reaction system, suggesting that the molecules of dissolved O_2_ consume the e^−^s to generate the ˙O_2_^−^ radicals, which further participate in Cr(vi) reduction. Further, the photocatalytic efficacy decreases from 84% to 78% soon after the employment of TBA, suggesting that ˙OH ions also make a minute involvement in the Cr(vi) reduction process. Thus, the above discussion informs that e^−^s and ˙O_2_^−^ are the dominant species engaged in Cr(vi) reduction.^48^ Further, the kinetics of various trapping reagents involved in the Cr(vi) reduction process tends to follow a pseudo-first-order model, as represented in Fig. 8b. Thereafter, the synthesized 2BrZn catalyst was successively used for multiple chromium reduction to verify its reusability and photo-stability. As shown in Fig. S6a,† the 2BrZn photocatalyst is stable up to 3 successive test runs. The reduction process was slightly reduced in the 4th test run and the 2BrZn photocatalyst shows 79% of photocatalytic activity, indicating the constancy feature of the catalyst for the photo-reduction process. The diffraction patterns perceived no substantial changes in photocatalyst after reaction and also the morphology of the used sample was quite intact with agglomeration verifying the photostability, as represented in Fig. S6b and c.†

**Fig. 9:**
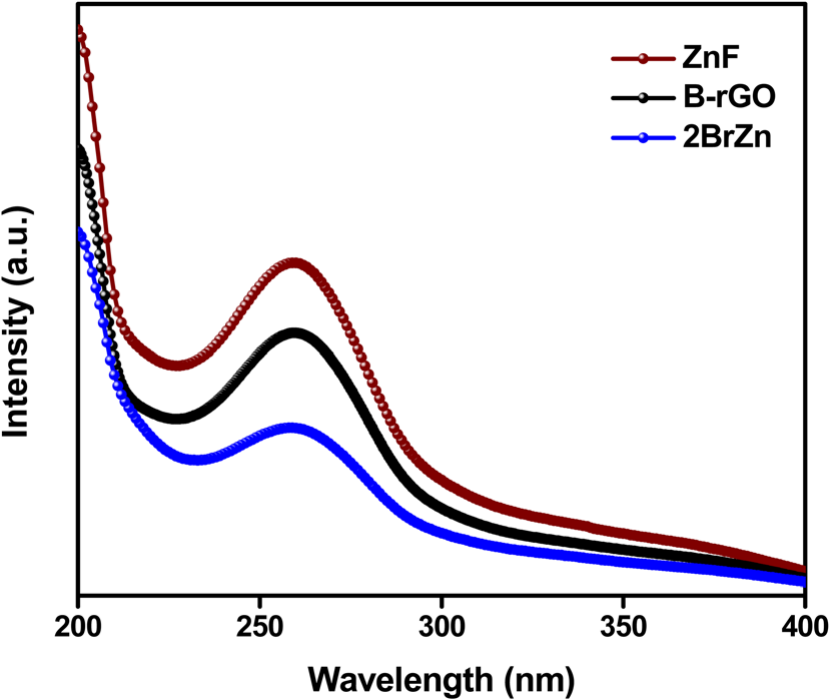
NBT experiments with ZnF, B-rGO and 2BrZn photocatalysts.

The production of ˙O_2_^−^ radicals was established by adopting NBT as the probing reagent. The Formazan signal was found to appear for all the specimens at 295 nm, confirming the materialization of ˙O_2_^−^ radicals (Fig. 9).^49^ As illustrated in the pictorial image, the intensity of the Formazan signal reduces on travelling from bare to composite materials in the order ZnF > B-rGO > 2BrZn, indicating that a large amount of ˙O_2_^−^ radicals are generated by the p–n-based S-scheme-oriented binary system.

### Photocatalytic charge transfer mechanism

**Fig. 10:**
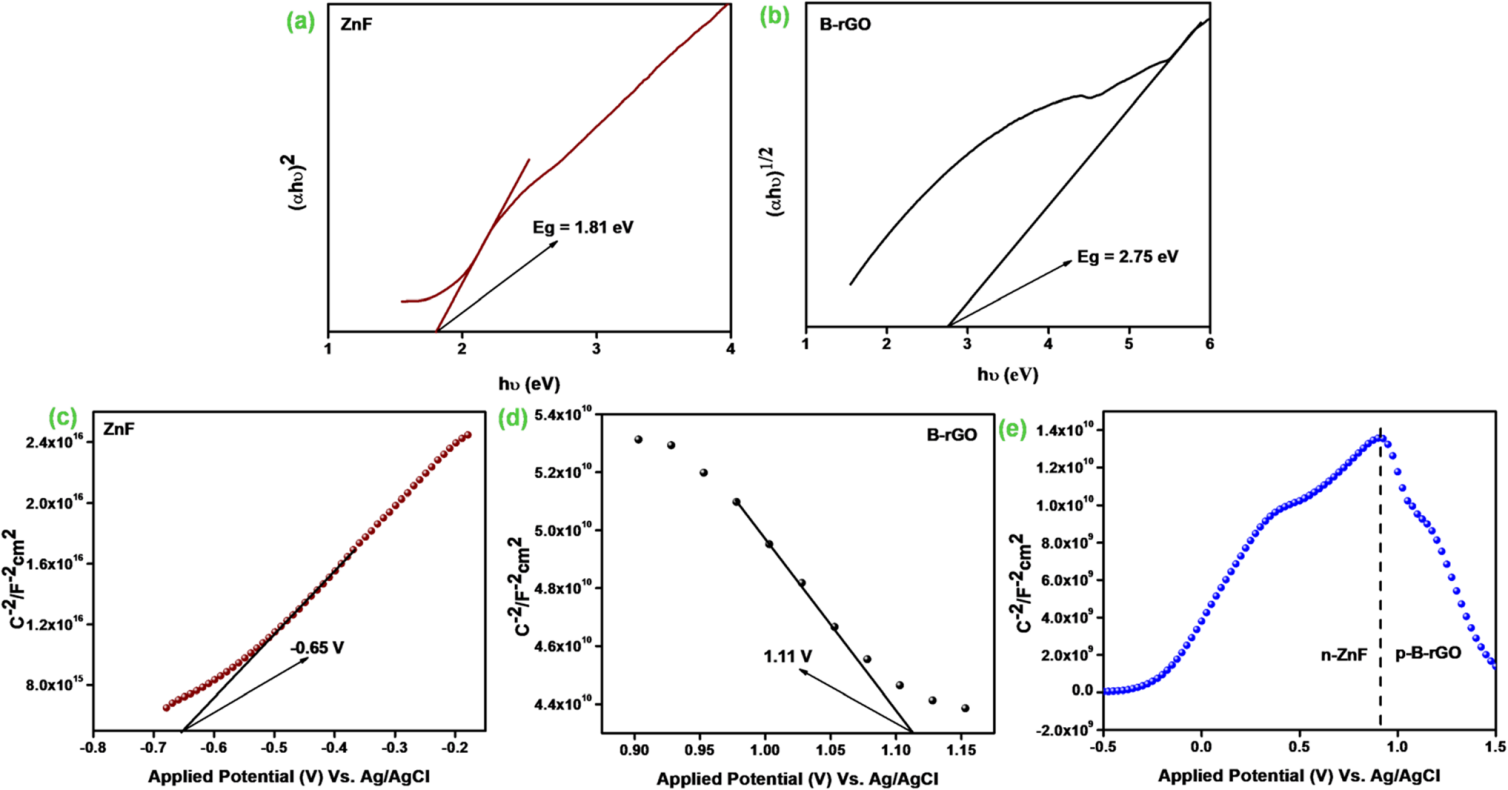
Band gap energy of (a) ZnF and (b) B-rGO. Mott–Schottky plots of (c) ZnF, (d) B-rGO, and (e) 2BrZn.

The band-gap energies (*E*_g_) of both B-rGO (Fig. 10a) and ZnF (Fig. 10b) parent materials were determined to be 2.75 and 1.81 eV respectively from the Tauc plot using the Kubelka–Munk expression, as provided in the following equation:^2^

10 *A*(*αhv*)^1/*n*^ = *A*(*hv* − *E*_g_)

We then performed the M–S experiment in order to collect valuable knowledge related to the type of semiconducting material and charge transfer across the electrolytic interface, and also to determine the exact band-edge potentials, as these were some of the essential parameters in describing the reaction mechanism. However, a negative slope was achieved for B-rGO representing the p-type feature of the semiconducting material with a band-edge potential of 1.11 V, as illustrated in Fig. 10c. Similarly, a positive slope was obtained for ZnF, indicating the n-type feature of the photocatalytic material with a flat-band of −0.65 V, as displayed in Fig. 10d. The flat band-potentials were converted to NHE scale by adopting the Nernst expression, as reported earlier in the literature.^16^ The *E*_CB_ and *E*_VB_ potentials of ZnF and B-rGO were assessed to be −0.04 and 1.77 eV *vs.* NHE, respectively. Further, combining the band-energy values obtained from the Tau plot, the VB and CB-edge positions of ZnF and B-rGO were found to be 1.7 and −1.05 eV respectively.

Notably, an inverted V-shaped current curvature was obtained for the 2BrZn composite specimen (Fig. 10e), further indicating that 2BrZn contains the properties of both p-type and n-type samples and the formation p–n heterojunctions at the interfacial region of ZnF and B-rGO components with exceptional electrical features.^17^ The fabrication of p–n junction at the interface results in the creation of an inner electric field at the meeting area of p- and n-type specimens, which eventually enhances the charge separation by permitting the photo-exciton to drift in both directions and also enables the smooth exciton distribution to the active site.^19^ Therefore, the fabricated specimen was found to be an essential photocatalytic material for heightened exciton segregation and prolonged life-time of e^−^/h^+^ pairs, which impacts the photocatalytic performances.

To have better understanding and knowledge regarding the Fermi-level alignment in the heterojunction material, the following expression was adopted to convert the NHE into the vacuum level: *E*_NHE_ = −4.5 − *E*_vac_.^17^ In this current study, the *E*_CB_ and *E*_VB_ values of ZnF were estimated to be −0.04 and 1.77 *vs.* vacuum level. Similarly, for B-rGO, the *E*_CB_ and *E*_VB_ values were evaluated to be −1.05 and 1.7 eV *vs.* vacuum level respectively. We know that *E*_F_ was situated at 0.1 eV beneath *E*_CB_ for the n-type material, whereas for the p-type semiconducting material, *E*_F_ has been positioned above *E*_VB_.^17^ The *E*_F_ values for n-type ZnF and p-type B-rGO were calculated to be respectively against the vacuum level. The evaluated Fermi position helps in determining the work-function using the following mathematical formula:^14^

11 *ϕA* = *E*_VAC_ − *E*_Fermi_

Therefore, the corresponding work-functions of ZnF and B-rGO were estimated to be 4.56 and 6.1 eV *vs.* vacuum respectively.

**Scheme 1:**
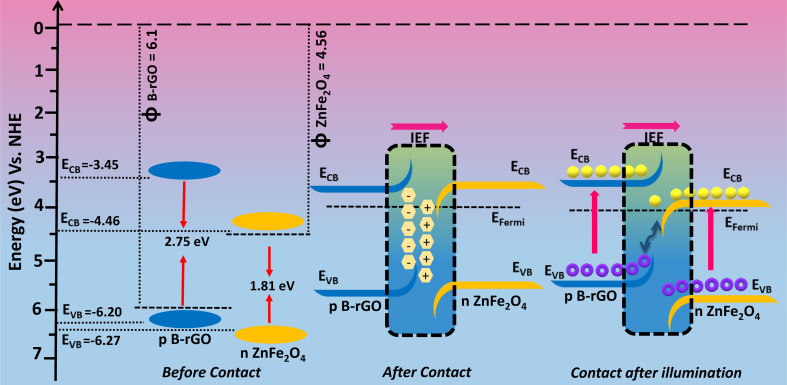
Visual representation of charge migration through the S-scheme in B-rGO/ZnF p–n heterostructures.

The e^−^s and ˙O_2_^−^ radicals were found to be the chief active species responsible for Cr(vi) reduction, as verified from the quenching experiments. As per the band-alignment analysis as discussed above, both ZnF and B-rGO exhibit a staggered type of alignment before interaction (Scheme 1), where *E*_CB_ and *E*_VB_ of ZnF were respectively positioned at +0.06 and 1.77 eV and those of B-rGO were located at −1.05 and 1.6 eV correspondingly. In staggered charge transfer mechanism, the photo-generated e^−^s trips from *E*_CB_ of B-rGO down to *E*_CB_ of ZnF and the h^+^ proceeds from *E*_VB_ of ZnF to *E*_VB_ of B-rGO and the shipment of excitons in this way could even extremely hamper Cr(vi) reduction. From the above discussion, it was noted that *E*_CB_ of ZnF was +0.06 eV, which does not match with the prerequisite potential for the conversion of dissolved O_2_ to ˙O_2_^−^ radicals and the reduction of Cr(vi) to Cr(OH)_3_ (−0.13 V *vs.* NHE).^49^ From the NBT experiments, it was observed that 2BrZn was able to produce a higher concentration of ˙O_2_^−^ radicals than the parent materials. Similarly, the maximum Cr(vi) reduction was obtained for 2BrZn, which signifies effective exciton separation across the interface. The generation of ˙O_2_^−^ radicals and photocatalytic performance signifies that the photo-generated charge-carrier follows an S-scheme charge-migration mechanism rather than the traditional type-II charge-transfer pathway. The scheme illustrates an imaginary p–n heterostructure-based S-scheme charge-transfer mechanism of 2BrZn heterojunction materials. The estimated band-edge potentials (*i.e.* conduction and valence band), *E*_F_ and work-functions of B-rGO and ZnF with respect to vacuum levels before contact are portrayed in Scheme 1 and Table 3. From Scheme 1, it can be observed that ZnF exhibits a lower work function than that of B-rGO and upon close interaction when a p–n heterojunction was formed in between ZnF and B-rGO, the negatively charged electrons will spontaneously surge from n-type ZnF to p-type B-rGO across the interface in order to achieve the Fermi-level equilibrium. After attaining the Fermi-level stability, an internal-electric field (IEF) was created at the interfacial junction of the 2BrZn composite. The e^−^s will flow from ZnF to B-rGO, resulting in the ZnF interface to be positively charged and B-rGO interface to be negatively charged. Under photon illumination, the internal-electric field drives the photo-generated e^−^s and h^+^ produced in the *E*_CB_ and *E*_VB_ positions of ZnF and B-rGO respectively. The IEF, band-edge bending, built-in electric-field at the interfacial space corridor and coulombic interaction were some crucial factors that drive the e^−^s from *E*_CB_ of ZnF across the interface and combines with h^+^ of *E*_VB_ of B-rGO in the manner of S-scheme charge transfer.^49^ In this way, the e^−^s at *E*_CB_ of B-rGO and h^+^ at *E*_VB_ of ZnF were preserved for photocatalytic reactions. The shortest exciton shipment pathway of *E*_CB_ of n-type ZnF to *E*_VB_ of p-type B-rGO also supports the S-scheme charge transmission phenomenon.

**Table 3:** CB, VB, *E*_Fermi_, *E*_Vac_ and work functions of ZnF and B-rGO samples

Material	CB	VB	*E*_Fermi_	*E*_Vac_	*ϕ* = 0–(*E*_Vac_)
ZnF	−0.04	1.77	+0.06	−4.56	4.56
B-rGO	−1.05	1.7	+1.6	−6.1	6.1

**Scheme 2:**
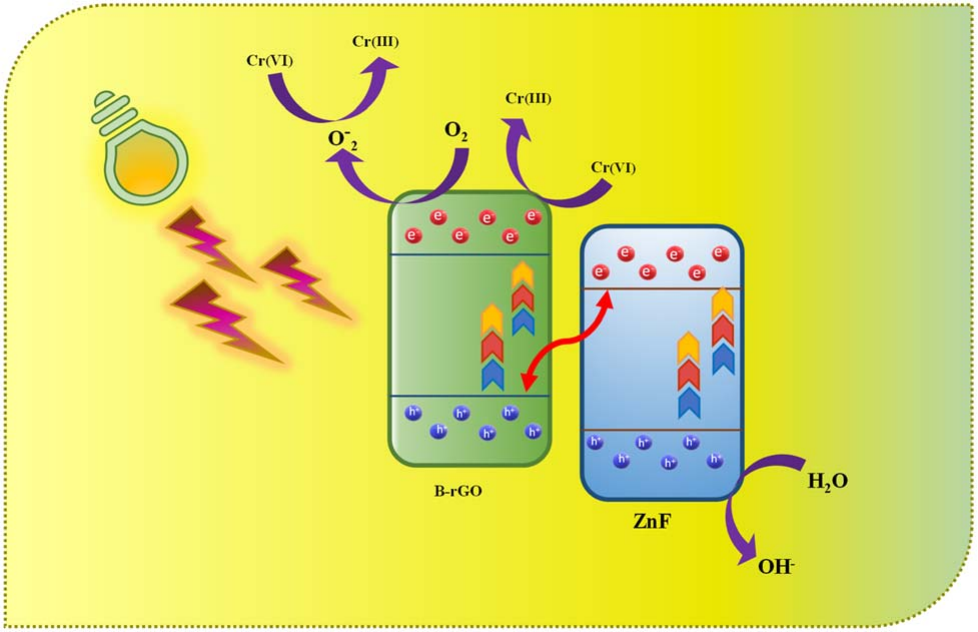
Schematic representation of photocatalytic Cr(vi) reduction by 2BrZn photocatalysts.

The possible photocatalytic mechanism for Cr(vi) reduction over 2BrZn composite materials is demonstrated in Scheme 2. Previously, it has been established that 2BrZn retains a p–n heterojunction, which follows an S-scheme charge migration mechanism. In detail, when the LED photon was illuminated on the photocatalytic material, the ZnF and B-rGO were stimulated to produce electrons, which jump to the CB leaving behind the holes on the VB. On the basis of well-matched band-alignment, the e^−^s residing at *E*_CB_ of ZnF travel to the interface of the heterojunction, and there they recombine with h^+^ in *E*_VB_ of B-rGO in an S-scheme charge transfer manner leaving behind the e^−^s and h^+^ free in *E*_CB_ and *E*_VB_ of B-rGO and ZnF respectively. The high-energy e^−^s on *E*_CB_ of B-rGO can convert O_2_ into ˙O_2_^−^, thereby involving in Cr(vi) reduction. The h^+^ existing on *E*_VB_ of ZnF oxidizes the OH^−^ ions to ˙OH radicals. *E*_CB_ of ZnF has a potential (+0.06 eV *vs.* NHE) which was lower than the potential of Cr(vi)/Cr(OH)_3_ (−0.13 V *vs.* NHE), and hence, Cr(vi) gets reduced to Cr(iii) by the e^−^s that exist on the CB of B-rGO.^48^ Therefore, the photo-reduction of Cr(vi) was arbitrated by the e^−^s and ˙O_2_^−^ radicals. It should be highlighted that the construction of a 2BrZn heterojunction may result in the development of more S-scheme heterojunctions that offer considerable active sites for Cr(vi) reduction.12
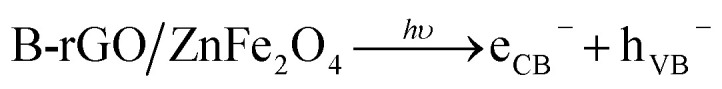

13 e_CB_^−^ + O_2_ → ˙O_2_^−^

14 CrO_4_^2−^ + 4H_2_O + 3e^−^ → Cr(OH)_3_ + 5OH^−^

15 CrO_4_^2−^ + H_2_O + ˙O_2_^−^ → Cr(OH)_3_ + 5OH^−^ + 3O_2_

## Conclusion

In conclusion, we have developed a novel B-rGO/ZnFe_2_O_4_ p–n heterojunction by adopting the most commonly utilized hydrothermal technique. The obtained B-rGO/ZnFe_2_O_4_ heterostructure serves as an S-scheme photocatalyst with significantly enhanced activity and stability. The photocatalytic activity of the heterojunction material was evaluated by Cr(vi) reduction and the optimized 2BrZn eradicates about 84% of Cr(vi) within 90 min under 20 W LED light illumination. The enhanced photocatalytic performance can be attributed to the effectual charge mobility with holes that regulates the charge separation process through S-scheme after the formation of p–n heterojunctions. Moreover, wide photon-absorption, strong-redox potential energy band and intimate interfacial contact are responsible for enhanced photocatalytic performance. The e^−^s and super-oxide were found to be the principal active species involved in Cr(vi) reduction, as verified by the trapping experiments. From the parameter study, it was observed that the existence of anionic CO_3_^2−^ and SO_4_^2−^ expressively hampers Cr(vi) eradication, whereas the cations have a minor effect. This work provides a feasible strategy for the fabrication of S-scheme photocatalysts based on p–n heterojunctions to address the environmental pollution problems.

## Conflicts of interest

There are no conflicts to declare.

## Supplementary Material

RA-014-D4RA03049D-s001
