# Supplementary material for: Facile fabrication of B-rGO/ZnFe2O4 p–n heterojunction-based S-scheme exciton engineering for photocatalytic Cr(vi) reduction: kinetics, influencing parameters and detailed mechanism
Source: RSC Adv. 2024 Jun 25;14(28):20312–27. doi: 10.1039/d4ra03049d (PMC11197843; doi:10.1039/d4ra03049d)
Supplement: RA-014-D4RA03049D-s001 [file RA-014-D4RA03049D-s001.pdf]

Electronic Supplementary Material (ESI) for RSC Advances.
This journal is © The Royal Society of Chemistry 2024

Fabrication of facile B-rGO/$ZnFe_2O_4$ p-n heterojunction based S-scheme Exciton Engineering for Photocatalytic Cr (VI) reduction: Kinetics, Influencing parameters and detailed mechanism.

Kundan Kumar Das[1], Upali Aparajita Mohanty[1], Lekha Paramanik[1], Dipti Prava Sahoo[1], and Kulamani Parida[1*]

[1]Centre for Nanoscience and Nanotechnology, SOA (Deemed to be University), Bhubaneswar-751030, Odisha (India)

[*]Corresponding author

E-mail: paridakulamani@yahoo.com &kulamaniparida@soa.ac.in

Tel. No. +91-674-2379425, Fax. +91-6 74-2581637

Characterization Techniques

The crystal structure and phase composition of the as-synthesized samples were investigated using X-ray diffraction (XRD) Rigaku Miniflex instrument (Cu Kα). The diffuse reflectance UV (DRUV) spectrum of the prepared samples was analyzed using JASCO-V750 UV−Vis spectrophotometer. The element distribution and morphology were revealed by FESEM with EDS system (JSM-7610F). The crystallographic nanoscale structure was determined using HR-TEM (JEOL-JEM-2100). The Fourier transform infrared spectroscopy (FT-IR) spectrum was verified by JASCO FTIR-4600 spectrometer. JASCO-FP-8300 fluorescence spectrometer was used for determining the PL spectra of the prepared samples. The electrochemical features of the synthesized specimens were investigated by a traditional three-electrode set-up containing 0.1 M Na_2SO_4 as electrolytic solution. The Pt is used as counter, Ag/AgCl is used as reference and B-rGO/ZnF used as the working electrodes respectively. A drop-casting method was implemented to prepare the working electrodes where nafion was mixed with slurry of photocatalyst and then deposited over the conducting FTO surface. The M-S studies of ZnF and 2BG/ZnF were conducted in a frequency of 500 Hz with amplitude of 0.025 mV. Similarly, the EIS measurement was done in the frequency range of 10^7-10^{-1} Hz with 0.01 mV amplitude. The transient photocurrent analysis (*I-t* curve) were done at 0.2V with two light "on" cycles under visible light irradiation and two light "off" cycles were conducted at every 30 sec time intermission. The photocurrent densities of were determined at a scan rate of 10 mV s^{-1} under visible light illumination. In the PEC investigation xenon lamp of 300W used as a light source attached with a 420 nm cutoff filter.

Photocatalytic Cr (VI) reduction

The Cr (VI) reduction was done to evaluate the photocatalytic supremacy of the synthesized samples. For Cr (VI) reduction, potassium dichromate solution was used as inspired wastewater for Cr (VI) pollutant. At first, 20 mg of photocatalyst was dispersed in 20 mL of potassium dichromate solution (20 ppm) and the suspension was stirred continuously for 30 min in dark to attain the adsorption-equilibrium, in order to eliminate the adsorption consequence. After that the suspension of pollutant and photocatalyst was continuously stirred under LED photons for 90 minutes. During photocatalytic reduction process, 5 mL of aliquot was extracted from the reaction suspension and the reduction of Cr (VI) to Cr (III) was quantified through DPC method, where Cr ion develops a purple color after reacting with DPC and sulfuric acid. Finally, the color-complex was measured by the UV-Vis absorbance at wavelength of 540 nm.[1] The efficiency of photocatalyst towards Cr (VI) reduction was estimated by using standard expression:

$$Cr\ (VI) reduction\ efficiency\ (\%) = \frac{C_0 - C_t}{C_0} * 100 \quad \text{……………..} (1)$$

Here, C_0 and C_t represents the initial concentration and concentration of Cr (VI) at time t.

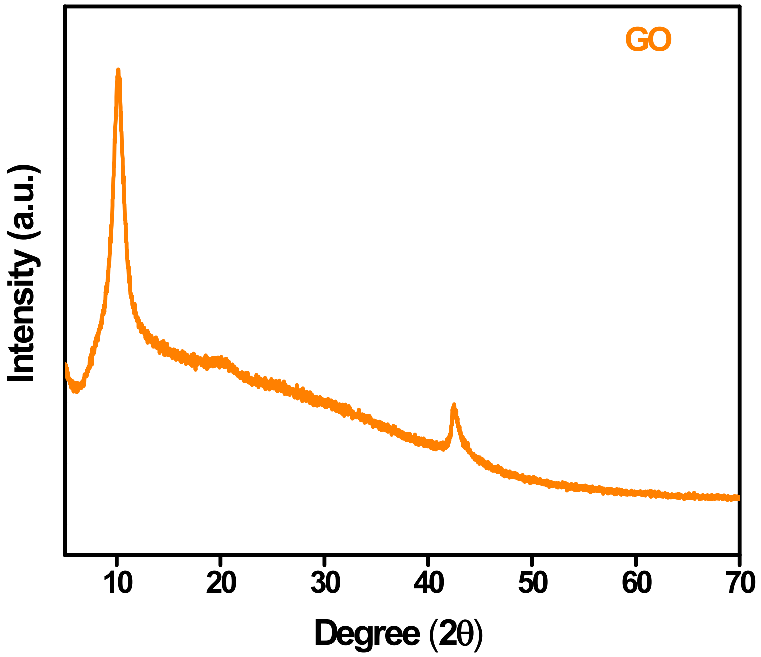

Fig. S1 XRD spectrum of GO

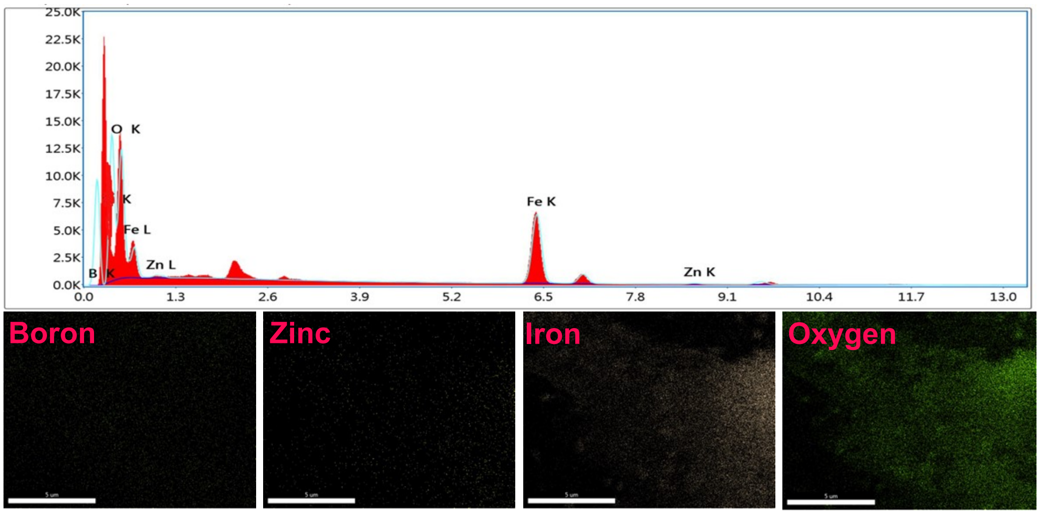

Fig. S2 EDAX and Elemental mapping of 2BrZn

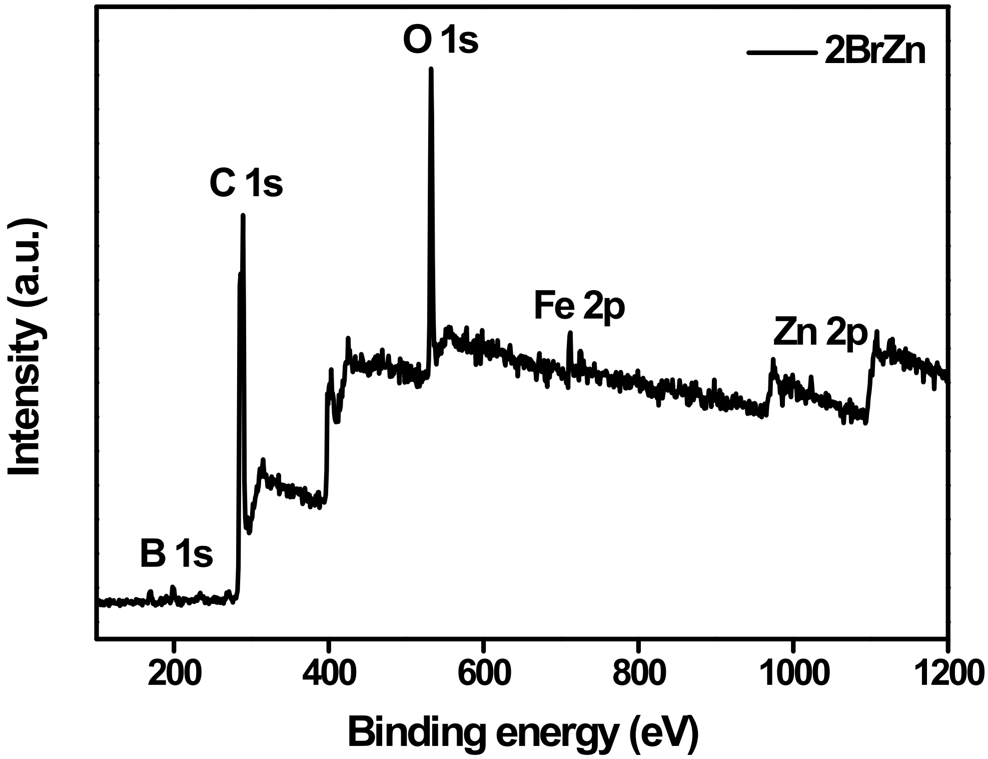

Fig. S3 Wide scan spectrum of 2BrZn

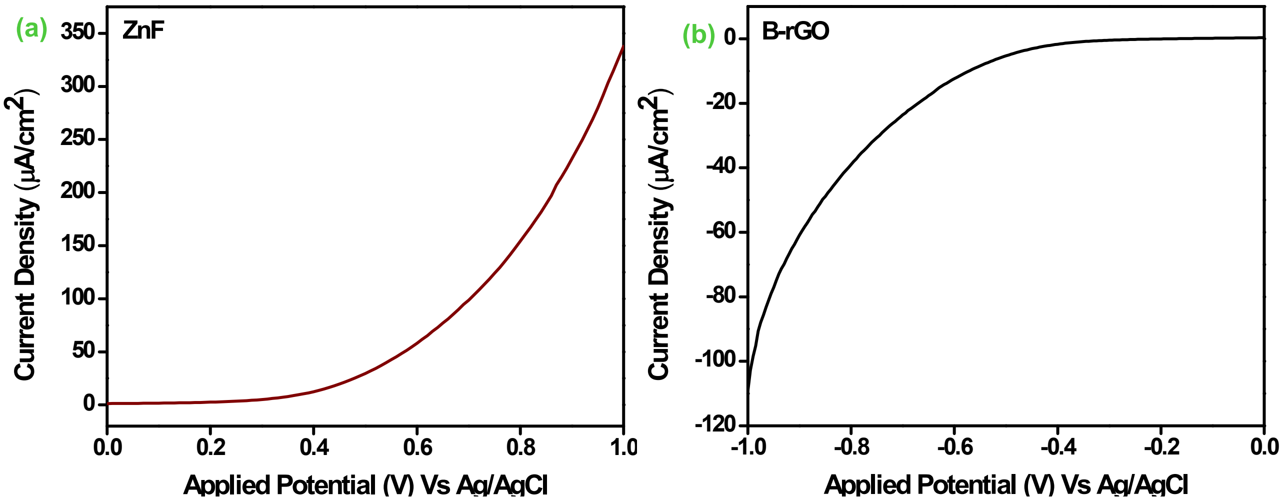

Fig. S4 Photocurrent density (a) ZnF and (b) B-rGO

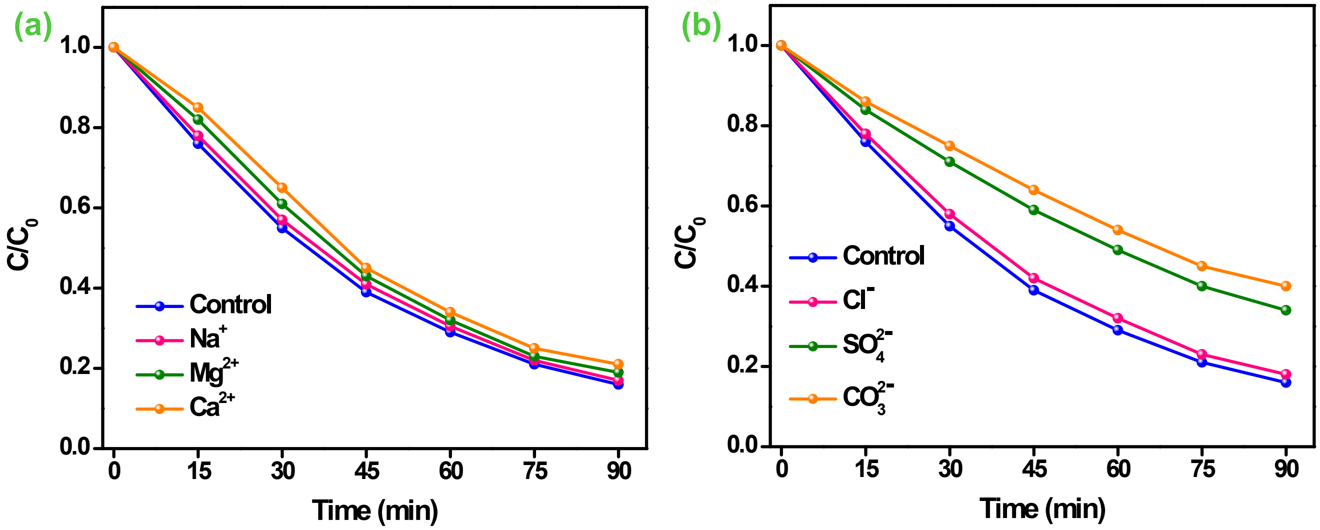

Fig. S5 Effect of operational parameters (a) Cations (b) anions over 2BrZn photocatalyst

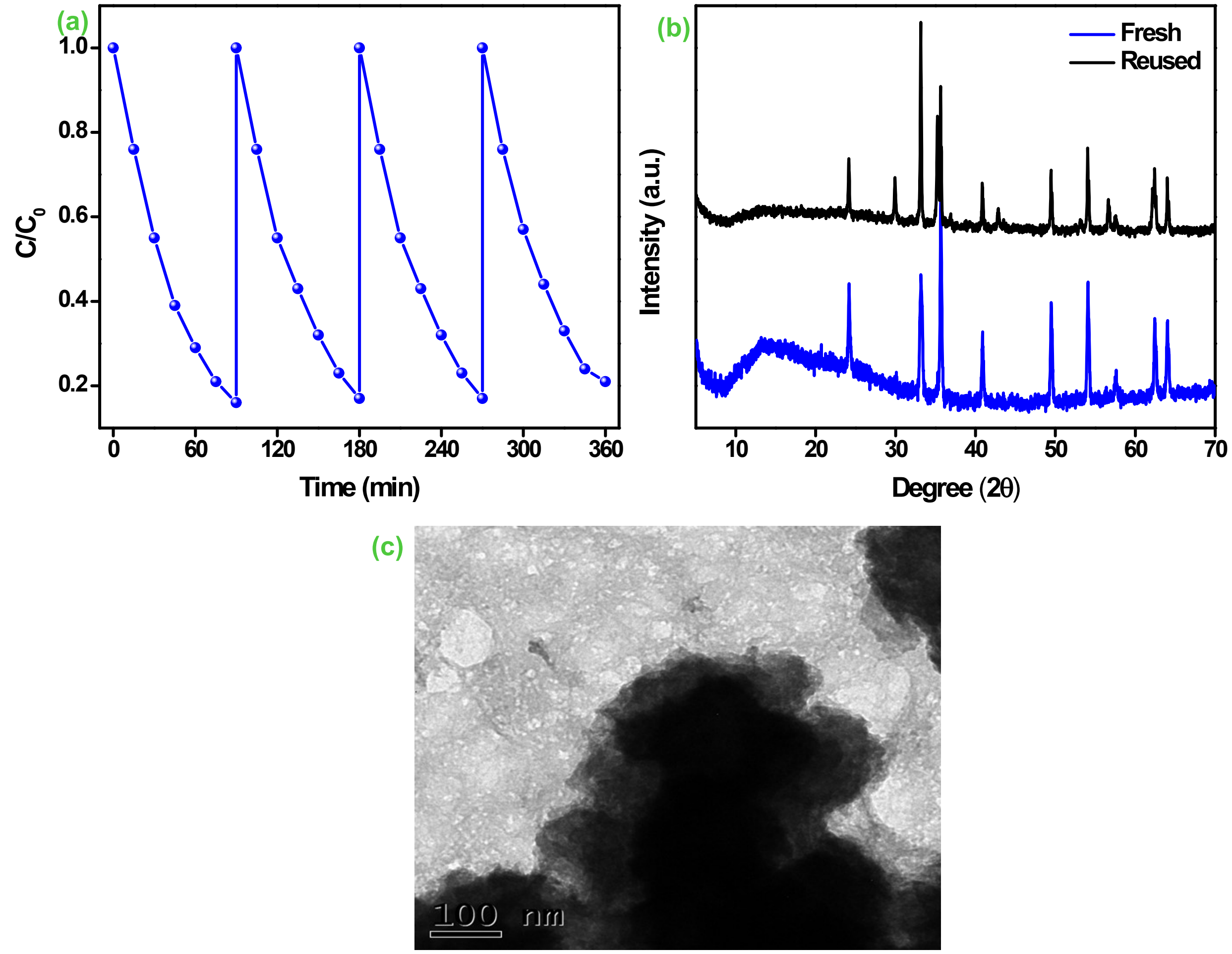

Fig. S6 Photostability test of 2BrZn (a) Cr (VI) reduction; (b) XRD image of before used and after used photocatalyst and (c) TEM image of after used photocatalyst

Table S1 Comparison of photocatalytic Cr (VI) reduction by various photocatalytic system with our system

**Photocatalysts**	**Light Source**	**Catalyst amount (mg)**	**pH**	**Cr (VI) mg/L**	**Irradiation (min)**	**Reduction Efficiency (%)**	**Ref.**
CQDs-TiO_{2-x} /rGO	300 W Xe lamp	100	5.68	10	80	80	[2]
TiO2 (P25)	LED Light	1000	3	10	120	60	[3]
ZFO@CNT	Visible	20	3	10	60	82	[4]
Reduced graphene oxide supported ZnO quantum dots	Visible	50	4	20	120	84	[5]
$ZnFe_2O_4/TiO_2$	200 W Tungsten Lamp	50	3	50	180	60	[6]
2BrZn	LED Light	20	3	20	90	84	This Work

References

1. D. K. Padhi, K. Parida, Facile fabrication of a-FeOOH nanorod/RGO composite: a robust photocatalyst for reduction of Cr(VI) under visible light irradiation, J. Mater. Chem. A, 2 (2014) 10300-10312. 10.1039/C4TA00931B
2. L. Xu, L. Yang, X. Bai, X. Du, Y. Wang, P. Jin, Persulfate activation towards organic decomposition and Cr(VI) reduction achieved by a novel CQDs-TiO_{2-x}/rGO nanocomposite, Chem. Eng. J. 373 (2019) 238–250. https://doi.org/10.1016/j.cej.2019.05.028
3.] Y. Li, Y. Bian, H. Qin, Y. Zhang, Z. Bian, Photocatalytic reduction behavior of hexavalent chromium on hydroxyl modified titanium dioxide, Appl. Catal. B 206 (2017) 293–299. https://doi.org/10.1016/j.apcatb.2017.01.044
4. A. Behera, S. Mansingh, K. K. Das, K. M. Parida, Synergistic $ZnFe_2O_4$-carbon allotropes nanocomposite photocatalyst for norfloxacin degradation and Cr (VI) reduction, J. Colloid Interface Sci. 544 (2019) 96-111. https://doi.org/10.1016/j.jcis.2019.02.056
5. K. V. Ashok Kumar, B. Lakshminarayana, D. Suryakala, Ch. Subrahmanyam, Reduced graphene oxide supported ZnO quantum dots for visible light-induced simultaneous removal of tetracycline and hexavalent chromium, RSC Adv. 10 (2020) 20494-20503. 10.1039/D0RA02062A.
6. G. Rekhila, M. Trari, Y. Bessekhouad, Characterization and application of the hetero-junction ZnFe2O4/TiO2 for Cr (VI) reduction under visible light, Appl. Water Sci. 7 (2017) 1273-1281. 10.1007/s13201-015-0340-9
